# Supplementary material for: Topological frustration of artificial spin ice
Source: Nat Commun. 2017 Jan 13;8:14009. doi: 10.1038/ncomms14009 (PMC5241825; doi:10.1038/ncomms14009)
Supplement: Supplementary Information — Supplementary Figures [file ncomms14009-s1.pdf]

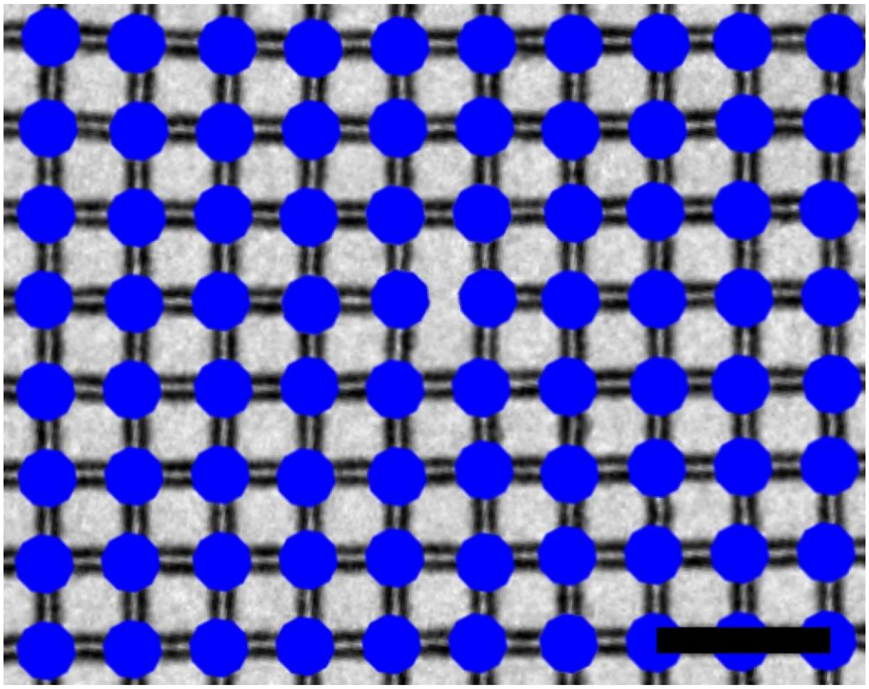

Supplementary Figure 1 | Vertex map of a non-topological defect. A simple vacancy, a single missing spin in an otherwise perfect square lattice, does not nucleate a vertex chain or result in topological frustration. Long range ground state ordering is not affected by the presence of a vacancy since it is not a topological defect. Scale bar is 1 μm.

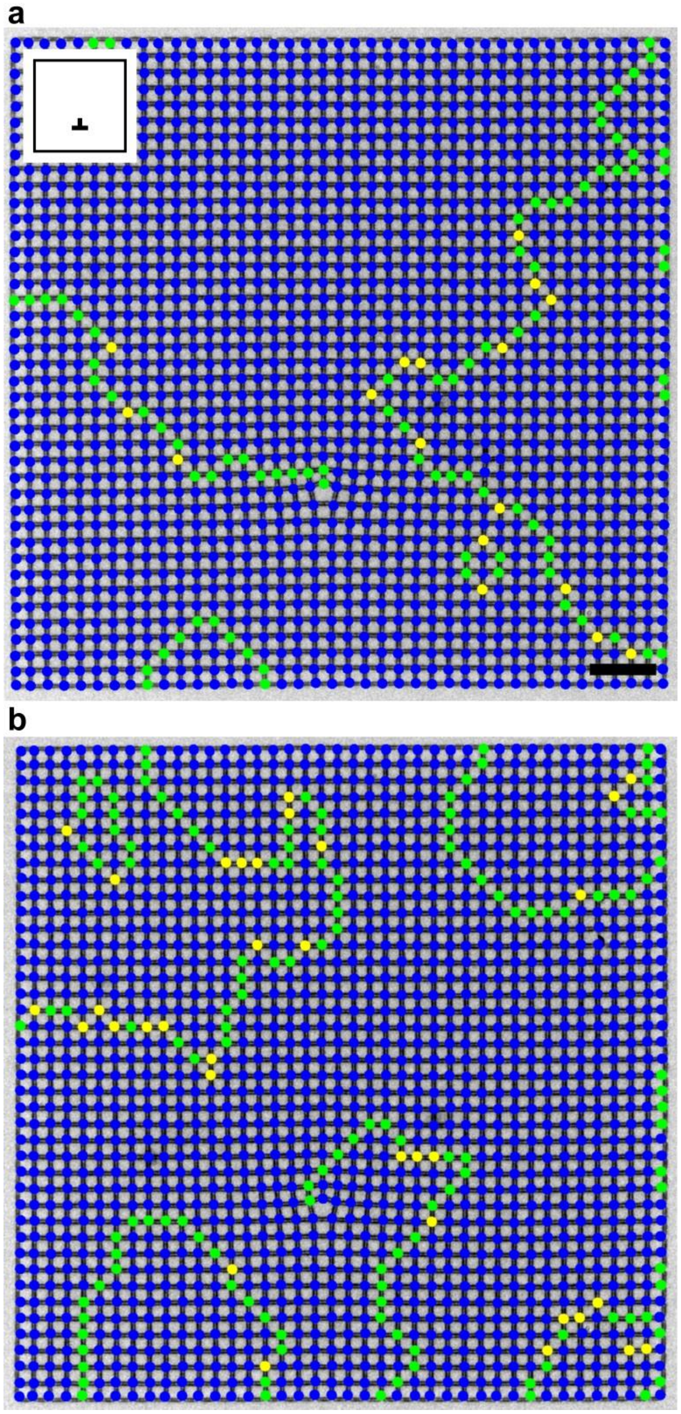

Supplementary Figure 2 | Repeated annealing of the same crystal. The same crystal is annealed twice under the same conditions and results in different domain configurations. The quenched disorder in the sample gives qualitatively similar, but not exactly reproducible ordering of vertices. Scale bar is 2 μm.

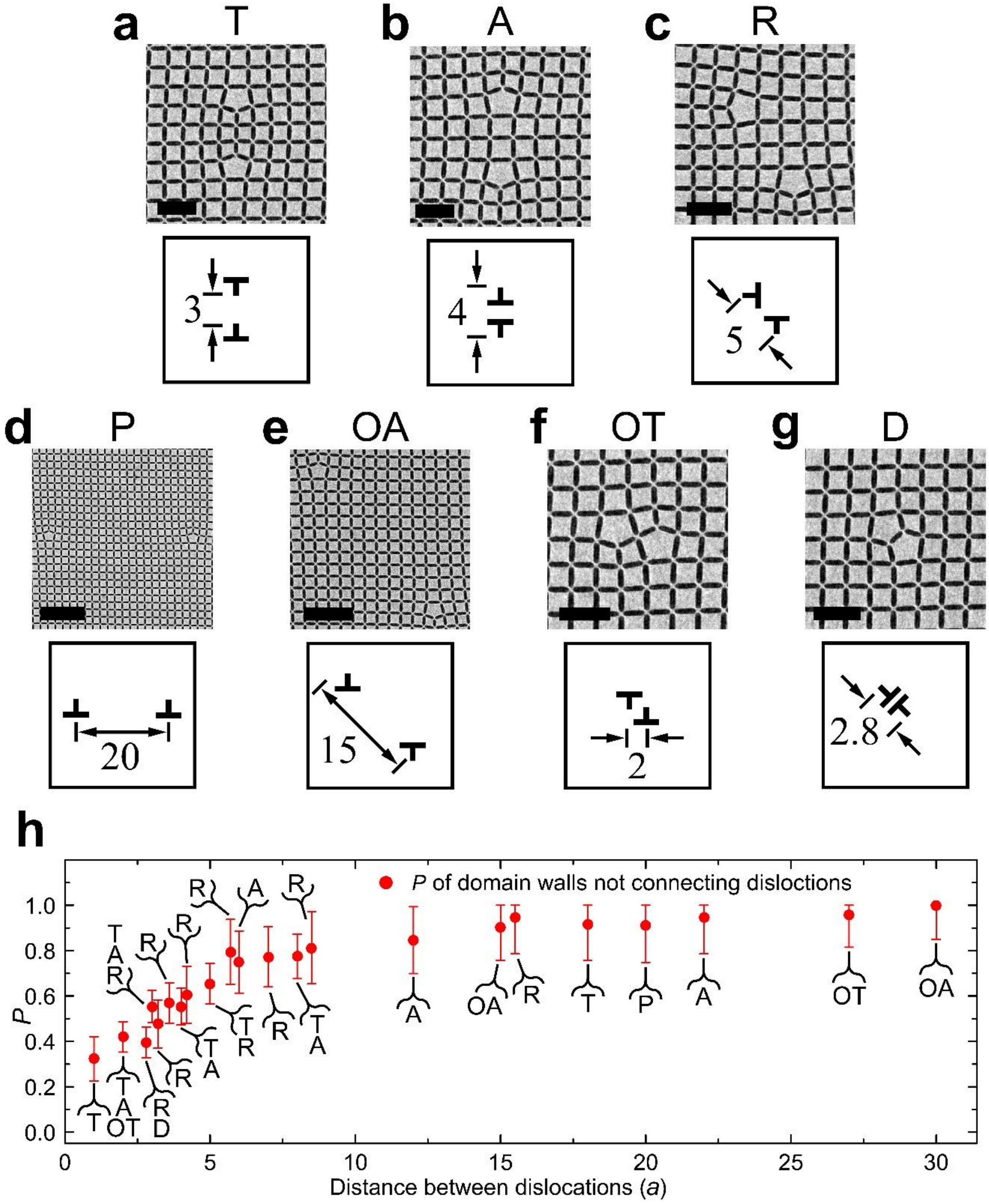

Supplementary Figure 3 | Geometries studied. (a-g) Definitions for families of two-dislocation geometries studied in this work. Each frame describes one family of geometries where the spacing between dislocations is varied. The spacing is given in units of the lattice constant (*a* ≈ 500 nm).

Each frame includes an in-focus TEM image of a representative crystal for that geometry and a schematic representation of the geometry. (**a**) Towards or T geometries have the two dislocations pointing at each other. Scale bar is 1 µm. (**b**) Away or A geometries have the two dislocations pointing away from each other. Scale bar is 1 µm. (**c)** Right or R geometries have the dislocations pointing at right angles to each other. T, A, and R are the most commonly studied geometries. Scale bar is 1 µm. (**d**) Parallel or P geometry has the dislocations pointing in the same direction. Scale bar is 3 µm. (**e**) Opposite-away or OA geometries have the dislocations pointing away from each other and offset. Scale bar is 2 µm. **(f**) Opposite-towards or OT geometries have the dislocations pointing towards each other and offset. Scale bar is 1 µm. (**g)** Diagonal or D geometry has the dislocations pointing diagonally with respect to the crystal axes. Scale bar is 1 µm. (**h**) Probability of domain walls not connecting dislocations vs. distance between dislocations in crystals with two topological defects. Same data as in Fig. 4 in the main text, but with annotations added indicating which geometries are included in each data point. Distances are expressed in units of the lattice constant $a \approx 500$ nm. Error bars are one standard deviation and are calculated from counting statistics. Each geometry includes approximately 40 crystals.
